# Temporal trend and spatial analysis of breast cancer mortality in Paraná, 2012-2021

**DOI:** 10.1590/S2237-96222025v34e20240171.en

**Published:** 2025-09-08

**Authors:** Sandy Gabrielle Pelegrini dos Santos, Carolina Fordellone Rosa Cruz, Kelly Holanda Prezotto, Ricardo Castanho Moreira, Maria José Quina Galdino, Rosana Rosseto Oliveira, Alessandro Rolim Scholze, Emiliana Cristina Melo

**Affiliations:** 1 Hospital de Amor, Programa de Residência em Atenção ao Câncer, Barretos, SP, Brazil; 2 Universidade Estadual do Norte do Paraná, Departamento de Enfermagem, Bandeirantes, PR, Brazil; 3 Universidade Estadual do Centro-Oeste, Programa de Pós-Graduação em Enfermagem em Atenção Primária à Saúde, Guarapuava, PR, Brazil; 4 Universidade Estadual do Norte do Paraná, Programa de Pós-Graduação em Enfermagem em Atenção Primária à Saúde Bandeirantes, PR, Brazil; 5 Universidade Estadual de Maringá, Programa de Pós-Graduação em Enfermagem Maringá, PR, Brazil

**Keywords:** Mortality, Breast Cancer, Women’s Health, Spatial Analysis, Time Series Studies, Mortalidad, Cáncer de Mama, Salud de la Mujer, Análisis Espacial, Estudios de Series Temporales

## Abstract

**Objectives:**

To analyze the temporal trend and identify spatial clusters of breast cancer mortality in Paraná state between 2012 and 2021.

**Methods:**

This was a time series study, with spatial analysis of breast cancer mortality rates in the 399 municipalities of Paraná. Data were selected from the Mortality Information System. Time series analysis was performed using the Prais-Winsten autoregression method. The Getis-Ord Gi* technique was used to identify spatial clusters.

**Results:**

A total of 8,819 breast cancer deaths were reported, with a rising trend in Paraná (annual percentage change [APC] +1.83; 95% confidence interval [95%CI] 0.86; 2.82). The East macro-region of the state stood out (APC +4.27; 95%CI 2.27; 6.32). Spatial analysis showed areas with a higher standardized mortality rate in the West and North macro-regions and areas with a lower rate in the East macro-region.

**Conclusion:**

In the period 2012-2021, there was an increase in breast cancer deaths in Paraná. Trend analysis showed that the phenomenon grew mainly in the East macro-region. In the spatial analysis, the West and North macro-regions had the highest concentration of the event. These results indicate areas in need of immediate intervention in the state.

## Resumen

**Objetivos:**

Analizar la tendencia temporal e identificar conglomerados espaciales de la mortalidad por cáncer de mama en Paraná entre 2012 y 2021.

**Métodos:**

Estudio de series temporales con análisis espacial de las tasas de mortalidad por cáncer de mama en los 399 municipios de Paraná. Los datos se seleccionaron del Sistema de Información de Mortalidad. El análisis de series temporales se realizó mediante el método de autorregresión de Prais-Winsten. Se empleó la técnica Getis-Ord Gi* para identificar conglomerados espaciales.

**Resultados:**

Se reportaron 8.819 muertes por cáncer de mama, con una tendencia creciente en Paraná (variación porcentual anual [VPA] +1,83; intervalo de confianza del 95% [IC95%] 0,86; 2,82). La macrorregión Este del estado se destacó (APC +4,27; IC95% 2,27; 6,32). El análisis espacial mostró áreas con una tasa de mortalidad estandarizada más alta en las macrorregiones Oeste y Norte, y áreas con una tasa más baja en la macrorregión Este.

**Conclusión:**

En el período 2012-2021, se observó un aumento de las muertes por cáncer de mama en Paraná. El análisis de tendencias mostró que el fenómeno se agravó principalmente en la macrorregión Este. En el análisis espacial, las macrorregiones con mayor concentración del evento corresponden al Oeste y al Norte. Estos resultados indican áreas que requieren intervención inmediata en el estado.

**Ethical aspects**

This research used public domain anonymized databases.

## Introduction

Breast cancer is a significant global public health problem, being the most common neoplasm and the leading cause of cancer mortality among women (1). In 2020, breast cancer was the most diagnosed carcinoma and had the highest mortality rate in the world, with estimates of 2.2 million cases (1) (79.7 cases per 100,000 women) (2) and 685,000 deaths that year (13.6 deaths per 100,000 women) (2). The estimate for 2025 is 768,533 breast cancer deaths globally (2). 

In Brazil, breast cancer is also the most common neoplasm in the female population, with an estimate of 74,000 new cases for the 2023-2025 three-year period, representing 66.54 cases per 100,000 women (3). The Southern region of Brazil is in fourth place among the country’s regions with the highest incidence, with an estimate of 11,230 new cases and risk of 41.06 cases per 100,000 women in 2023, following the Southeast region (52.83 per 100,000 female inhabitants), the Midwest region (47.31 per 100,000 female inhabitants) and the Northeast region (42.11 per 100,000 female inhabitants) (3).

Specifically in relation to the Southern region of Brazil, Paraná ranks third among the region’s three states, with an incidence rate of 41.0 per 100,000 women for each year of the 2023-2025 three-year period (3) and a mortality rate of 12.3 per 100,000 women in 2021 (4). 

Given the magnitude of this form of cancer and the overloading of the Brazilian National Health System due to the increase in hospitalizations, high-cost treatments and high mortality (5), the Ministry of Health has been implementing public health policies throughout Brazil, aiming to reduce breast cancer incidence and mortality (6). 

With regard to addressing this neoplasm, there are challenges, such as sociodemographic and territorial barriers, which impede access to diagnosis and treatment of the disease in the Brazilian states, including Paraná. These barriers include vulnerabilities inherent to social class, place of residence and level of schooling (6).

Analysis of the temporal trend of breast cancer deaths is of utmost importance, since this form of cancer is highly preventable (7). Risk factors for its development, such as smoking, obesity, physical inactivity and alcohol consumption, can be the object of intervention through public actions aimed at raising public awareness and addressing such risk factors (8). 

This study aimed to analyze the temporal trend and identify spatial clusters of breast cancer mortality in Paraná between 2012 and 2021.

## Methods

### Design

This was a time series study of breast cancer mortality in women living in the 399 municipalities of Paraná, based on Mortality Information System data.

### Setting

**Figure 1:**
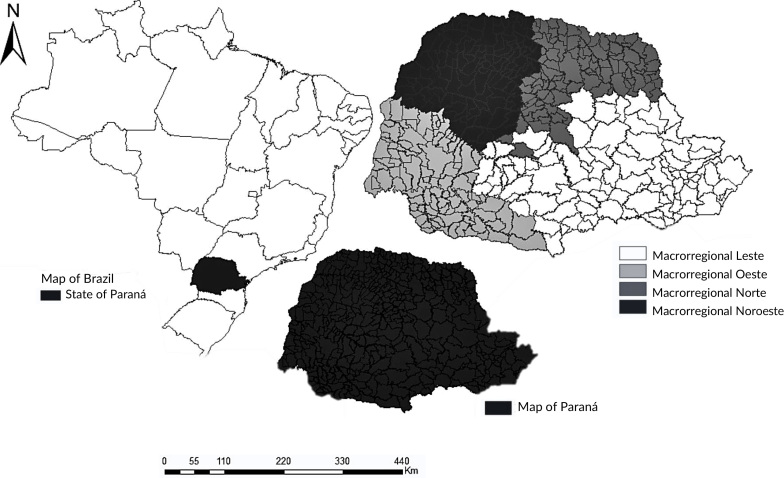
Health macro-regions. Paraná, 2024

Paraná is located in the Southern region of Brazil. It is considered the most populous state in the region, with an estimated population of 11,443,208 inhabitants in 2023. It has a territorial area of 199,298,981 km^2^, administratively divided into four health macro-regions (Northwest – 115 municipalities, West – 94 municipalities, North – 97 municipalities and East – 93 municipalities) and 22 health regions (9) (Figure 1).

### Study participants

The study included all women aged 20-80 years or older who died from breast cancer, residing in the 399 municipalities of Paraná. These deaths were reported on the Mortality Information System between 2012 and 2021.

### Study variables

For the purposes of data analysis, the following variables were included: year of death (2012-2021), age group (in years: 20-49; 50-69; 70-80 or over), race/skin color (White, non-White), marital status (has partner, no partner) and schooling (in years of study: 0-7, 8 or more). Deaths from breast cancer in men were excluded from the study. Unknown or unreported variables were not considered in the analysis. The 15-19 years age group had no reported breast cancer deaths up until to the time the data source was accessed.

### Data sources and measurement

The mortality information we analyzed was derived from the Mortality Information System, using code C50 of the tenth revision of the International Classification of Diseases (ICD-10) in order to select deaths. Population information (number of women residing in each municipality by age group) was extracted from the data source entitled Resident population – a study of population estimates by municipality, age and sex 2000-2021 – Brazil. Both sources of information were accessed via the Brazilian National Health System Information Technology Department (10) in March 2023. Regional information (cartographic base of municipalities) for the spatial analysis of breast cancer deaths was obtained in April 2023 via the Brazilian Institute of Geography and Statistics, using the scale 1:250,000 – version 2022 (11).

### Bias control

In order to minimize the impact on the statistical analyses of mortality, the number of deaths was corrected using the methodology proposed by the World Health Organization in 2002 (12). This methodology proportionally redistributes the number of deaths from ill-defined causes among deaths from breast cancer.

The procedure resulted in the correction percentage for ill-defined causes and the correction factor for each municipality and year, using the following equations.

- The correction percentage for ill-defined causes is equal to the total number of female deaths, minus deaths from external causes, divided by the total number of female deaths, minus deaths from external causes, minus deaths from ill-defined causes.
-
$\text{Correction factor}=1+\frac{\left(\text{correction percentage for ill-defined causes}-1\right)}{2}$

The correction factor was then multiplied by the total number of breast cancer deaths, thus obtaining corrected deaths due to ill-defined causes. To compose the ill-defined causes and external causes, female deaths grouped in ICD-10 chapters XVIII and XX (signs, symptoms and abnormal clinical and laboratory findings and external causes of morbidity and mortality) were used. 

The corrected information was grouped and tabulated using Microsoft Excel 2021. The crude mortality rate was calculated by dividing the number of deaths by the number of female residents, multiplied by 100,000 inhabitants. After exploratory analysis of the data, we decided to calculate the age-standardized rates, using the direct method (13), to be used in the spatial distribution and temporal trend of the rates by health macro-region.

### Statistical methods

Time series analysis was performed using the Prais-Winsten autoregression method (14). The dependent variable considered was the breast cancer mortality rate, while the independent variable was the year. This trend was presented as annual percentage change (APC) with a 95% confidence interval (95%CI) and could be classified as stationary (when zero was part of the 95%CI), rising (when the 95%CI values were positive) or falling (when the 95%CI values were negative) (14). The analyses were performed using Stata 14.

In order to identify the spatial distribution of breast cancer deaths, we used the cartographic database of the municipalities. The freely accessible Google Earth tool was used to obtain the geographic coordinates of each municipality. All deaths were georeferenced according to the reporting municipality, without the need for exclusion.

We performed spatial analysis of deaths and mortality rates by municipality. We applied the Getis-Ord Gi* technique to identify spatial clusters, whereby a z-score is generated for municipalities that are statistically significant. The higher the z-score, the more intense the clustering of high values or hot spots, indicating greater occurrence of the event. In the case of a negative z-score, clusters of low values or cold spots indicated lower occurrence of the event (15).

In addition to the z-score, the p-value and significance level (Gi-Bin) were also obtained, which identified statistically significant hot and cold spots. Values of +/- 3 reflected statistical significance at the 99% confidence level, +/- 2 at the 95% confidence level, and +/- 1 at the 90% confidence level, with zero corresponding to areas that were not statistically significant (15).

Arcgis version 10.5 was used for this analysis.

### Data access and cleaning methods

The data were accessed via the on-line databases of the Mortality and Resident Population Information System – a study of population estimates by municipality, age and sex 2000-2021 – Brazil, made available via the Brazilian National Health System Information Technology Department platform (http://www2.datasus.gov.br/DATASUS/index.php?%20area=0203) (10) and via the Brazilian Institute of Geography and Statistics website (https://www.ibge.gov.br/geociencias/cartas-e-mapas/bases-cartograficas-continuas.html) (11), excluding unknown or unreported data.

## Results

Between 2012 and 2021, there were 8,425 breast cancer deaths among women living in Paraná, with an overall rate of 20.3 deaths per 100,000 women. Correction of the number of deaths increased the overall magnitude of the rates, as the number of deaths increased to 8,819, and the overall rate to 29.3 deaths per 100,000 women.

The highest breast cancer mortality rates occurred among women in the 50-69 years age group (9.8 per 100,000 women), those of White race/skin color (17.9 per 100,000 women), those with no partner (11.1 per 100,000 women) and those with 0-7 years of schooling (12.0 per 100,000 women) (Table 1).

**Table 1:** Distribution of demographic characteristics of deaths, temporal trend, annual percentage change (APC) and 95% confidence interval (95%CI) of crude breast cancer mortality rates (per 100,000) in women. Paraná, 2012-2021

Variable	Number of deaths	Mortality rate	APC	95%CI	p-value	Trend
**Age group** (**in years**)						
20-49	2,015	4.8	-0.33	-1.36; 0.72	0.490	Stationary
50-69	4,092	9.8	2.09	0.94; 3.25	0.003	Rising
70-80 or over	2,713	6.5	3.22	1.95; 4.51	<0.001	Rising
**Race/skin color**						
White	7,427	17.9	1.37	0.17; 1.57	0.029	Rising
Non-White	1,393	3.3	4.32	2.80; 5.86	<0.001	Rising
Unknown	233					
**Marital status**						
No partner	4,602	11.1	2.88	0.43; 3.86	<0.001	Rising
Has partner	4,217	10.1	0.73	-0.57; 2.05	0.234	Stationary
Unknown	211					
**Schooling** (**in years of study**)						
0-7	5,003	12.0	-1.27	-23.06; 0.06	0.059	Stationary
8 or more	3,816	9.2	6.17	4.84; 7.54	<0.001	Rising
Unknown	467					
Total	8,819	29.3	1.83	0.86; 2.82	<0.001	Rising

The overall temporal trend of crude breast cancer mortality rates in Paraná was rising (APC +1.83; 95%CI 0.86; 2.82). For almost all variables analyzed, the trend was also rising, with the exception of the 20-49 years age group (APC -0.33; 95%CI -1.36; 0.72), having a partner (APC 0.73; 95%CI -0.57; 2.05) and 0-7 years of schooling (APC -1.27; 95%CI -23.06; 0.06), for which the trend was stationary (Table 1).

The East and North macro-regions had the highest overall age-standardized mortality rates. In the East macro-region, there were 31.2 deaths per 100,000 women, and in the North macro-region, 28.7 per 100,000 women. The highest age-standardized mortality rates were observed in the 70-80 years or over age group (36.0 per 100,000 women in the East macro-region), among those of White race/skin color (19.6 per 100,000 women in the East macro-region) and those with 0-7 years of schooling (13.0 per 100,000 women in the North macro-region). The not having a partner marital status variable had higher rates in the East (12.2 per 100,000 women) and Northwest (10.6 per 100,000 women) macro-regions (Table 2).

**Table 2:** Age-standardized breast cancer mortality rate (per 100,000) in women, by macro-region. Paraná, 2012-2021

Variables	East Rate=31.2		West Rate=27.0		North Rate=28.7		Northwest Rate=27.7	
	n	Rate	n	Rate	n	Rate	n	Rate
**Age group** (**in years**)								
20-49	987	5.3	331	1.2	369	1.9	328	1.6
50-69	2,042	24.7	648	5.5	722	8.4	679	7.8
70-80 and over	1,381	36.0	398	10.4	488	17.2	446	15.6
**Race/skin color**								
White	3,884	19.6	1,188	16.4	1,293	17.4	1,063	15.2
Non-white	526	2.6	189	2.6	287	3.8	391	5.6
Unknown	195		11		20		7	
**Marital status**								
No partner	2,431	12.2	640	8.8	785	10.5	746	10.6
Has partner	1,979	9.9	737	10.2	794	10.7	707	10.1
Unknown	211							
**Schooling** (**in years**)								
0-7	2,257	11.4	930	12.8	968	13.0	848	12.1
8 or more	2,153	10.8	447	6.6	611	8.2	605	8.6
Unknown	229		67		125		46	

**Figure 2:**
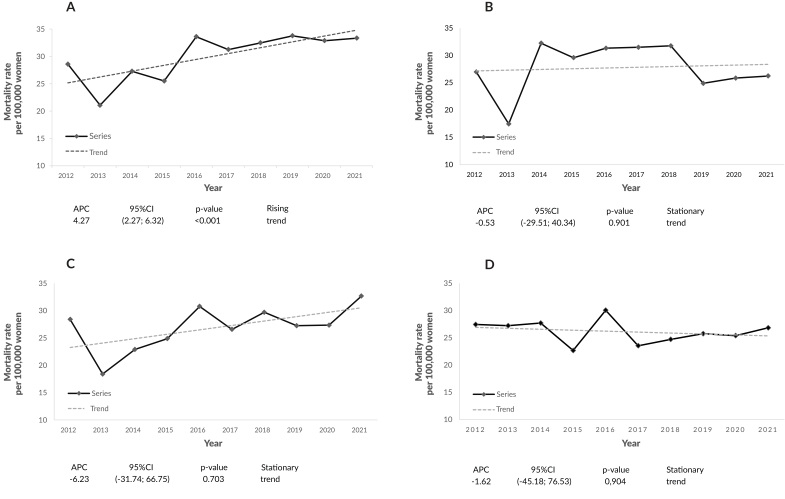
Time series of breast cancer mortality rates (per 100,000) in women, standardized by age in the health macro-regions: (A) East; (B) North; (C) Northwest; (D) West. Paraná, 2012-2021

Breast cancer mortality rates by macro-region showed an increasing trend in the East macro-region (APC +4.27; 95%CI 2.27;6.32) (Figure 2A). The North (APC -0.53; 95%CI -29.51; 40.34), Northwest (APC -6.23; 95%CI -31.74; 66.75) and West (APC -1.62; 95%CI -45.18; 76.53) macro-regions showed a stationary trend (Figures 2B, 2C and 2D).

**Figure 3:**
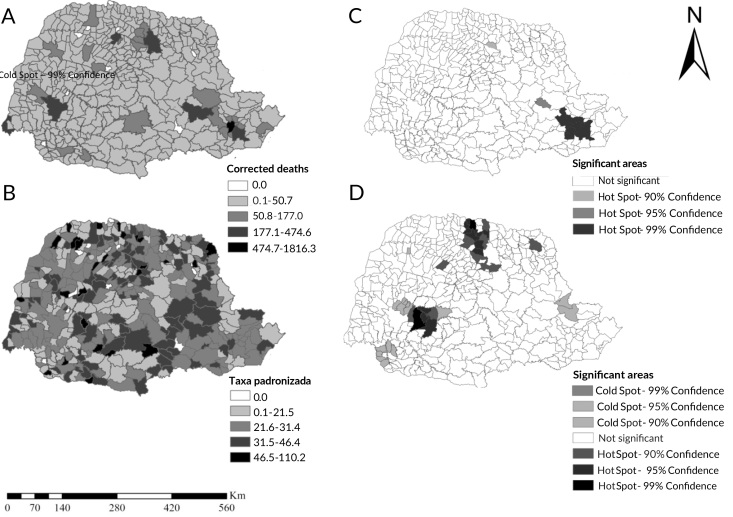
Spatial distribution of age-standardized breast cancer deaths and mortality rates and their risk areas; (A) spatial distribution of corrected numbers of deaths; (B) spatial distribution of age-standardized breast cancer mortality rates; (C) spatial areas of highest occurrence referring to the corrected numbers of deaths from breast cancer; (D) spatial areas of highest occurrence referring to age-standardized breast cancer mortality rates. Paraná, 2012-2021

The East macro-region showed hot spots (municipalities with the highest incidence) mainly involving the municipalities of the Curitiba metropolitan region (p-value <0.001; z-score 4.76) (Figure 3C). The West macro-region presented hot and cold spots (municipalities with lower occurrences) in relation to spatial clusters considering mortality rates (Figure 3D). The North macro-region showed hot spots, while the East macro-region showed cold spots. These data presented a p-value of 0.745 and a z-score of -0.32.

## Discussion

Between 2012 and 2021, there was a higher mortality rate among women aged 50-69, of predominantly White race/skin color, with no partner and with up to 7 years of schooling, with a rising temporal trend for the whole of Paraná, especially for the East macro-region. Spatial distribution by number of deaths also identified hot spots (clusters of high values) in the East macro-region. Distribution by mortality rates showed hot and cold spots in the West macro-region, hot spots in the North macro-region and cold spots in the East macro-region.

The limitations of this study were the possible existence of underreporting on the Mortality Information System. In addition, there is a possibility of bias regarding the updating of data on the system over time, after analysis of cases by epidemiological surveillance teams. These limitations were minimized by the use of the correction factor applied in this study. Moreover, it needs to be considered that, in the period 2020-2022, affected by the COVID-19 pandemic, there were limitations regarding breast cancer screening, favoring the possibility that some COVID-19 deaths could have been potentially caused by breast cancer in other circumstances.

The breast cancer mortality rate in Paraná was higher than in other regions of the country. In the state of Bahia, the mortality rate was 9.3 deaths per 100,000 women in 2018 (16). In the state of São Paulo, the estimated rate for 2021 was 11.71 deaths per 100,000 women (4).

The upward trend in breast cancer mortality rates in Paraná can also be seen in other Brazilian states. In Santa Catarina, there was a significant increase of 8.38% in 2017 (17). In Ceará, there was an annual increase of 0.43% in reported deaths during the period 2005-2015 (18). However, this increase did not occur only in Brazil. In seventeen Latin America and Caribbean countries between 1997 and 2017, there was an annual increase of 2.8% in breast cancer mortality in women (19).

The increase in breast cancer mortality may be related to health service inefficiency, specifically the lack of prophylactic measures, early diagnosis and treatment in the early stages of the disease (18). Among the biggest problems faced by Primary Health Care are the underfunding of the Brazilian National Health System, spending freezes and service deterioration. These issues have resulted in failures in care and, consequently, in increased mortality (20).

Analyzing the demographic characteristics of the population studied, a scenario with a rising trend and high mortality was observed in the 50-69 years age group, coinciding with the target population for screening. In order to achieve significant reductions in breast cancer mortality, the World Health Organization stipulated that 70.0% of the female population belonging to the target age group for screening should undergo mammography. However, in 2022, Brazil only screened 65.9% of these women (4), which may be a factor contributing to the high mortality and rising trend in this age group.

A rising temporal trend was also seen in women aged 70-80 years and over. This scenario can be explained by the presence of comorbidities in this age group, such as diabetes, high blood pressure, depression and arthritis. Such comorbidities result in lower survival rates for these women, as they minimize the intensity of the treatment to be implemented (21).

Although the highest mortality occurs among women over 50 years of age, deaths also occur among those aged 20-49 years. There is a need for health education to raise women’s awareness about their breasts, clinical examination performed by a health professional and early screening for younger women with a family history of the disease (22).

In this analysis, women of White race/skin color showed a rising trend and high mortality rates, which may be related to the fact that a large part of the population of Paraná (70.32%) identifies as White (23). Although the highest mortality rates were found among those of White race/skin color, there was also an increase in those of non-White race/skin color, which demonstrates women’s vulnerability to breast cancer regardless of race/skin color.

In the state of Sergipe, between 1996 and 2017, higher mortality rates and a rising temporal trend were observed among women who did not have a partner (24). Marital status is not considered a risk factor for breast cancer, but it is understood that this characteristic is important for assessing the social profile of these women. This occurs because it is an indicator of vulnerability, since women with no partner are more likely to be deprived of the financial and emotional support that a partner could give, which makes access to diagnosis and early treatment difficult, corroborating mortality from this cause (25).

The higher breast cancer death rates and the stationary trend among women with up to seven years of schooling are also confirmed by other findings. In the state of Rio Grande do Sul, between 1999 and 2019, it was found that women with less than eight years of schooling had higher mortality rates (26). Data from 2019 showed that low schooling is one of the characteristics for diagnosis in advanced stages of the disease due to seeking health care late (27). 

The stationary trend of this variable highlighted the need for paying greater attention to women with less than seven years of schooling, using mechanisms that they can understand and encourage screening. However, women with eight or more years of schooling showed a rising trend, which indicated the need to intensify timely and organized screening through mammography, early diagnosis and identification of signs and symptoms of breast changes regardless of schooling (22).

In the spatial analysis, the East macro-region had the highest rates and a rising temporal trend, which may be associated with the fact that this macro-region is home to 30.9% of the state’s residents, being the most populous region, in which the highest mortality rates due to chronic non-communicable diseases in Paraná are also found (28). These findings suggest difficulties in accessing and offering health services to combat these diseases, which may be contributing to the increase in breast cancer mortality in this region.

When analyzing the spatial distribution (GI*) of breast cancer deathsrft, it can be seen that in the East macro-region there were hot spots in the Curitiba metropolitan region, Paraná’s state capital, the most populous area of the state (28), which is also where there were municipalities with the highest number of deaths, thus explaining the predominance of deaths in this region. This argument includes the large number of people in peripheral areas, who have difficulty in accessing health services and prophylactic measures, which in turn favors delay in diagnosis and early treatment (29).

Analysis of the spatial distribution (GI*) of breast cancer mortality rates revealed hotspots in the North and West macro-regions, indicating a concentration of municipalities with higher breast cancer mortality rates. The municipalities that make up these macro-regions have a municipal human development index of 0.4 to <0.6 and 0.6 to <0.8, considered low-medium and medium. These parameters indicate social and economic inequality, already known to hinder the population’s access to health services (28), proven in this study by the high concentrations of municipalities located in this macro-region with the highest mortality rates.

Between 2012 and 2021, there was an increase in breast cancer deaths in Paraná. According to the trend analysis, this phenomenon was rising, especially for women over 50 years of age, without partners and regardless of schooling and race/skin color. The main areas with an increasing trend, and consequently with a greater need for immediate intervention, are the municipalities of the East macro-region.

Primary care services should plan awareness-raising activities and provide emergency care, including flexible hours that allow women to attend outside their working hours in order to undergo clinical examinations performed by health professionals. There is a need to educate women about the signs and symptoms of breast cancer so as to promote self-awareness among women regarding possible changes in their breasts, encouraging them to seek immediate imaging screening.

The results of this study are important for planning and implementing public health actions and policies aimed at preventing and detecting breast cancer early, thus contributing to reducing mortality from this cause.

## Data Availability

The database and the analysis codes used in this research are available from: http://www2.datasus.gov.br/DATASUS/index.php?%20area=0203; and https://www.ibge.gov.br/geociencias/cartas-e-mapas/bases-cartograficas-continuas.html.
