# Supplementary material for: Temporal trend and spatial analysis of breast cancer mortality in Paraná, 2012-2021
Source: Epidemiol Serv Saude. 2025 Sep 8;34:e20240171. doi: 10.1590/S2237-96222025v34e20240171.en (PMC12435970; doi:10.1590/S2237-96222025v34e20240171.en)
Supplement: Translation

# Translation: Tendência temporal e análise espacial da mortalidade por câncer de mama no Paraná, 2012-2021

**DOI:** 10.1590/S2237-96222025v34e20240171.pt

**Authors:** Sandy Gabrielle Pelegrini dos Santos, Carolina Fordellone Rosa Cruz, Kelly Holanda Prezotto, Ricardo Castanho Moreira, Maria José Quina Galdino, Rosana Rosseto Oliveira, Alessandro Rolim Scholze, Emiliana Cristina Melo

**Affiliations:** 1 Hospital de Amor, Programa de Residência em Atenção ao Câncer, Barretos, SP, Brasil; 2 Universidade Estadual do Norte do Paraná, Departamento de Enfermagem, Bandeirantes, PR, Brasil; 3 Universidade Estadual do Centro-Oeste, Programa de Pós-Graduação em Enfermagem em Atenção Primária à Saúde, Guarapuava, PR, Brasil; 4 Universidade Estadual do Norte do Paraná, Programa de Pós-Graduação em Enfermagem em Atenção Primária à Saúde Bandeirantes, PR, Brasil; 5 Universidade Estadual de Maringá, Programa de Pós-Graduação em Enfermagem Maringá, PR, Brasil

**Keywords:** Mortalidade, Câncer de Mama, Saúde da Mulher, Análise Espacial, Estudos de Séries Temporais

## Abstract

**Objetivos:**

Analisar a tendência temporal e identificar aglomerados espaciais da mortalidade por câncer de mama no Paraná entre 2012 e 2021.

**Métodos:**

Tratou-se de estudo de séries temporais, com análise espacial das taxas de mortalidade por câncer de mama nos 399 municípios do Paraná. Os dados foram selecionados do Sistema de Informações sobre Mortalidade. A análise de séries temporais foi realizada por meio do método de autorregressão Prais-Winsten. Para identificar aglomerados espaciais, utilizou-se a técnica Getis-Ord Gi*.

**Resultados:**

Foram notificados 8.819 óbitos por câncer de mama, com tendência crescente no Paraná (variação percentual anual [VPA] +1,83; intervalo de confiança 95% [IC95%] 0,86; 2,82). Destacou-se a macrorregional Leste (VPA +4,27; IC95% 2,27; 6,32). A análise espacial mostrou áreas de maior taxa padronizada de mortalidade nas macrorregionais Oeste e Norte e áreas de menor taxa na macrorregional Leste.

**Conclusão:**

No período 2012-2021, houve aumento dos óbitos por câncer de mama no Paraná. A análise de tendência evidenciou que o fenômeno cresceu principalmente na macrorregional Leste. Na análise espacial, as macrorregionais com maior concentração do evento envolvem a Oeste e a Norte. Esses resultados sinalizam áreas com necessidade de intervenção imediata no estado.

**Aspectos éticos**

Esta pesquisa utilizou bancos de dados de domínio público e anonimizados.

## Introdução

O câncer de mama constitui um significativo problema mundial de saúde pública, sendo a neoplasia mais incidente e a principal causa de mortalidade por câncer entre mulheres (1). Em 2020, o câncer de mama foi o carcinoma mais diagnosticado e de maior taxa de mortalidade no mundo, com estimativas de 2,2 milhões de casos (1) (79,7 casos a cada 100 mil mulheres) (2) e 685 mil óbitos no ano (13,6 óbitos por 100 mil mulheres) (2). Para 2025, estimam-se 768.533 óbitos por câncer de mama no mundo (2). 

No Brasil, o câncer de mama também é a neoplasia mais incidente na população feminina, com estimativa de 74 mil casos novos para o triênio de 2023-2025, o que representa 66,54 casos por 100 mil mulheres (3). O Sul do país ocupa a quarta posição entre as regiões de maior incidência, onde estimou-se 11.230 casos novos com o risco de 41,06 casos por 100 mil mulheres em 2023, ficando atrás somente das regiões Sudeste (52,83 por 100 mil mulheres habitantes), Centro-Oeste (47,31 por 100 mil mulheres habitantes) e Nordeste (42,11 por 100 mil mulheres habitantes) (3). 

Na região Sul do país, o Paraná ocupa a terceira posição entre os estados da região, apresentando incidência de 41,0 por 100 mil mulheres para cada ano do triênio 2023-2025 (3) e taxa de mortalidade de 12,3 por 100 mil mulheres em 2021 (4). 

Em razão da magnitude apresentada por esse câncer e pela sobrecarga do Sistema Único de Saúde com o aumento de internações, os tratamentos de alto custo e a alta mortalidade (5), o Ministério da Saúde vem empregando políticas públicas de saúde no país, no intuito de diminuir a incidência e a mortalidade ocasionadas por essa doença (6). 

Quando se trata do enfrentamento dessa neoplasia, há desafios, a exemplo das barreiras sociodemográficas e territoriais, que impedem o acesso ao diagnóstico e ao tratamento da doença nos estados do país, inclusive no Paraná. Tais barreiras abrangem vulnerabilidades inerentes à classe social, ao local de residência e ao nível de escolaridade (6).

A análise da tendência temporal dos óbitos por câncer de mama é de suma importância, visto que tal câncer é altamente prevenível (7). Os fatores de risco para o desenvolvimento dessa neoplasia, como o tabagismo, a obesidade, a inatividade física e o consumo de álcool, podem ser intervencionados por meio de ações públicas voltadas à conscientização populacional e ao combate a tais fatores de risco (8). 

Este estudo teve como objetivos analisar a tendência temporal e identificar aglomerados espaciais da mortalidade por câncer de mama no Paraná entre 2012 e 2021.

## Métodos

### Delineamento

Tratou-se de estudo de séries temporais da mortalidade por câncer de mama em mulheres residentes nos 399 municípios do Paraná, com dados do Sistema de Informações sobre Mortalidade. 

### Contexto

O Paraná está localizado no Sul do Brasil. É considerado o estado mais populoso da região, com população estimada em 11.443.208 habitantes em 2023. Tem área territorial de 199.298.981 km^2^, dividido administrativamente em quatro macrorregionais de saúde (Noroeste – 115 municípios, Oeste – 94 municípios, Norte – 97 municípios e Leste – 93 municípios) e 22 regionais de saúde (9) (Figura 1).

**Figura 1:** Macrorregionais de saúde. Paraná, 2024

### Participantes do estudo

A população do estudo incluiu todos os óbitos por câncer de mama em mulheres entre 20-80 anos ou mais de idade, residentes nos 399 municípios do Paraná. Esses óbitos foram notificados no Sistema de Informações sobre Mortalidade no período 2012-2021. 

### Variáveis do estudo

Para a análise dos dados, incluíram-se as seguintes variáveis: ano dos óbitos (2012-2021), faixa etária (em anos: 20-49; 50-69; 70-80 ou mais), raça/cor da pele (branca, não branca), situação conjugal (com companheiro, sem companheiro) e escolaridade (em anos de estudo: 0-7, 8 ou mais). Os óbitos por câncer de mama em homens foram excluídos do estudo. Variáveis ignoradas ou não informadas não foram consideradas na análise. A faixa etária 15-19 anos não apresentou nenhum óbito por câncer de mama até o momento do acesso à fonte de dados.

### Fontes de dados e mensuração

As informações de mortalidade analisadas são derivadas do Sistema de Informações sobre Mortalidade, utilizando-se o código C50 da décima revisão da Classificação Internacional de Doenças (CID-10) para seleção dos óbitos. As informações populacionais (número de mulheres residentes em cada município por faixa etária) foram extraídas de População residente – estudo de estimativas populacionais por município, idade e sexo 2000-2021 – Brasil. Ambas as fontes de informações foram acessadas por meio do Departamento de Informática do Sistema Único de Saúde (10) em março de 2023. As informações regionais (base cartográfica dos municípios) para a análise espacial dos óbitos por câncer de mama foram obtidas por meio do Instituto Brasileiro de Geografia e Estatística, utilizando a escala 1:250.000 – versão 2022 (11) em abril de 2023.

### Controle de viés

Visando minimizar o comprometimento das análises estatísticas de mortalidade, foi realizada a correção dos números de óbitos por meio da metodologia proposta pela Organização Mundial da Saúde em 2002 (12). Essa metodologia redistribui proporcionalmente o número de óbitos por causa mal definida entre os óbitos por câncer de mama. 

O procedimento resultou no percentual de correção por causa mal definida e no fator de correção por cada município e ano, utilizando as equações a seguir.

- O percentual de correção por causa mal definida é igual ao total de óbitos femininos, menos os óbitos por causas externas, dividido pelo total de óbitos femininos, menos os óbitos por causas externas, menos os óbitos por causa mal definida.
-
$\text{Fator de correção}=1+\frac{\left(\text{percentual de correção por causa mal definida}-1\right)}{2}$

Em seguida, multiplicou-se o fator de correção pelo total de óbitos por câncer de mama, obtendo os óbitos corrigidos por causa mal definida. Para compor as causas mal definidas e causas externas, foram utilizados os óbitos femininos agrupados nos capítulos XVIII e XX da CID-10 (sinais, sintomas e achados anormais de exames clínicos e de laboratório e causas externas de morbidade e mortalidade). 

As informações corrigidas foram agrupadas e tabuladas no Microsoft Excel 2021. Calculou-se a taxa bruta de mortalidade dividindo o número de óbitos pelo número de mulheres residentes, multiplicado por 100 mil habitantes. Após a análise exploratória dos dados, optou-se por calcular as taxas padronizadas por idade, por meio do método direto (13), para serem utilizadas na distribuição espacial e tendência temporal das taxas por macrorregionais de saúde. 

### Métodos estatísticos

A análise de séries temporais foi realizada por meio do método de autorregressão Prais-Winsten (14). A variável dependente considerada foi a taxa de mortalidade por câncer de mama, e a variável independente, o ano. Tal tendência foi apresentada como variação percentual anual (VPA) com intervalo de confiança de 95% (IC95%) podendo ser classificada como estacionária (quando o IC95% incluiu o valor zero), crescente (quando os valores do IC95% eram positivos) ou decrescente (quando os valores do IC95% eram negativos) (14). As análises foram realizadas no software Stata 14.

Para a identificação da distribuição espacial dos óbitos por câncer de mama, foi utilizada a base cartográfica dos municípios. Utilizou-se a ferramenta Google Earth de acesso livre para a obtenção das coordenadas geográficas de cada município. Todos os óbitos foram georreferenciados de acordo com o município de notificação, sem necessidade de exclusão.

Procedeu-se à análise espacial dos óbitos e das taxas de mortalidade por município. Para identificar aglomerados espaciais, foi aplicada a técnica Getis-Ord Gi*, na qual é gerado um z-escore para municípios que são estatisticamente significativos. Quanto maior o z-escore, mais intenso é o agrupamento de valores altos ou pontos quentes, mostrando maior ocorrência do evento. Para o z-escore negativo, considerou-se o agrupamento de baixos valores ou pontos frios, mostrando menor ocorrência do evento (15). 

Além do z-escore, o p-valor e o nível de significância (Gi-Bin) também foram fornecidos, os quais identificaram pontos quentes e frios estatisticamente significativos. Valores de +/- 3 refletiram a significância estatística com o nível de confiança de 99%, +/- 2 com o nível de confiança de 95% e +/- 1 com o nível de confiança 90%, sendo o valor zero correspondente às áreas não estatisticamente significativas (15).

Para esta análise, foi utilizado o software Arcgis versão 10.5.

### Acesso aos dados e métodos de limpeza

Os dados foram acessados via internet nas bases de dados do Sistema de Informações sobre Mortalidade e de População residente – estudo de estimativas populacionais por município, idade e sexo 2000-2021 – Brasil, disponibilizados pela plataforma Departamento de Informática do Sistema Único de Saúde (http://www2.datasus.gov.br/DATASUS/index.php?%20area=0203) (10) e pela página eletrônica do Instituto Brasileiro de Geografia e Estatística (https://www.ibge.gov.br/geociencias/cartas-e-mapas/bases-cartograficas-continuas.html) (11), excluídos os dados ignorados ou não informados.

## Resultados

No período 2012-2021, ocorreram 8.425 óbitos por câncer de mama em mulheres residentes no Paraná, com taxa geral de 20,3 óbitos por 100 mil mulheres. A correção do número de óbitos elevou a magnitude geral das taxas, já que o número de óbitos passou para 8.819, e a taxa geral, para 29,3 óbitos por 100 mil mulheres. 

As maiores taxas de mortalidade por câncer de mama ocorreram entre mulheres com faixa etária de 50-69 anos (9,8 por 100 mil mulheres), brancas (17,9 por 100 mil mulheres), sem companheiro (11,1 por 100 mil mulheres) e com 0-7 anos de estudo (12,0 por 100 mil mulheres) (Tabela 1).

**Tabela 1:** Distribuição das características demográficas dos óbitos, tendência temporal, variação percentual anual (VPA) e intervalo de confiança de 95% (IC95%) das taxas brutas de mortalidade por câncer de mama (por 100 mil) em mulheres. Paraná, 2012-2021

Variável	Número de óbitos	Taxa de mortalidade	VPA	IC95%	p-valor	Tendência
**Faixa etária** (**em anos**)						
20-49	2.015	4,8	-0,33	-1,36; 0,72	0,490	Estacionária
50-69	4.092	9,8	2,09	0,94; 3,25	0,003	Crescente
70-80 ou mais	2.713	6,5	3,22	1,95; 4,51	<0,001	Crescente
**Raça/cor da pele**						
Branca	7.427	17,9	1,37	0,17; 1,57	0,029	Crescente
Não branca	1.393	3,3	4,32	2,80; 5,86	<0,001	Crescente
Ignorados	233					
**Situação conjugal**						
Sem companheiro	4.602	11,1	2,88	0,43; 3,86	<0,001	Crescente
Com companheiro	4.217	10,1	0,73	-0,57; 2,05	0,234	Estacionária
Ignorados	211					
**Escolaridade** (**em anos**)						
0-7	5.003	12,0	-1,27	-23,06; 0,06	0,059	Estacionária
8 ou mais	3.816	9,2	6,17	4,84; 7,54	<0,001	Crescente
Ignorados	467					
Total	8.819	29,3	1,83	0,86; 2,82	<0,001	Crescente

A tendência temporal geral das taxas brutas de mortalidade por câncer de mama no Paraná foi crescente (VPA +1,83; IC95% 0,86; 2,82). Para quase todas as variáveis analisadas, a tendência também foi crescente, com exceção da faixa etária de 20-49 anos (VPA -0,33; IC95% -1,36; 0,72), situação conjugal com companheiro (VPA 0,73; IC95% -0,57; 2,05) e escolaridade de 0-7 anos de estudo (VPA -1,27; IC95% -23,06; 0,06), para as quais a tendência foi estacionária (Tabela 1). 

As macrorregionais Leste e Norte apresentaram as maiores taxas gerais de mortalidade padronizadas por idade. Na macrorregional Leste, ocorreram 31,2 óbitos por 100 mil mulheres, e na macrorregional Norte, 28,7 por 100 mil mulheres. As maiores taxas de mortalidade padronizadas por idade foram observadas para a faixa etária de 70-80 ou mais anos (36,0 por 100 mil mulheres na macrorregional Leste), a raça/cor da pele branca (19,6 por 100 mil mulheres na macrorregional Leste) e a escolaridade 0-7 anos (13,0 por 100 mil mulheres na macrorregional Norte). A variável situação conjugal sem companheiro apresentou maiores taxas nas macrorregionais Leste (12,2 por 100 mil mulheres) e Noroeste (10,6 por 100 mil mulheres) (Tabela 2).

**Tabela 2:** Taxa de mortalidade por câncer de mama (por 100 mil) em mulheres padronizada por idade, segundo macrorregionais. Paraná, 2012-2021

Variáveis	Leste Taxa=31,2		Oeste Taxa=27,0		Norte Taxa=28,7		Noroeste Taxa=27,7	
	n	Taxa	n	Taxa	n	Taxa	n	Taxa
**Faixa etária** (**em anos**)								
20-49	987	5,3	331	1,2	369	1,9	328	1,6
50-69	2.042	24,7	648	5,5	722	8,4	679	7,8
70-80 e mais	1.381	36,0	398	10,4	488	17,2	446	15,6
**Raça/cor da pele**								
Branca	3.884	19,6	1.188	16,4	1.293	17,4	1.063	15,2
Não branca	526	2,6	189	2,6	287	3,8	391	5,6
Ignorados	195		11		20		7	
Situação conjugal								
Sem companheiro	2.431	12,2	640	8,8	785	10,5	746	10,6
Com companheiro	1.979	9,9	737	10,2	794	10,7	707	10,1
Ignorados	211							
**Escolaridade** (**em anos**)								
0-7	2.257	11,4	930	12,8	968	13,0	848	12,1
8 ou mais	2.153	10,8	447	6,6	611	8,2	605	8,6
Ignorados	229		67		125		46	

As taxas de mortalidade por câncer de mama por macrorregional evidenciaram tendência de crescimento na macrorregional Leste (VPA +4,27; IC95% 2,27;6,32) (Figura 2A). As macrorregionais Norte (VPA -0,53; IC95% -29,51; 40,34), Noroeste (VPA -6,23; IC95% -31,74; 66,75) e Oeste (VPA -1,62; IC95% -45,18; 76,53) apresentaram tendência estacionária (Figuras 2B, 2C e 2D).

**Figura 2:** Série temporal das taxas de mortalidade por câncer de mama (por 100 mil) em mulheres, padronizadas por idade nas macrorregionais de saúde: (A) Leste; (B) Norte; (C) Noroeste; (D) Oeste. Paraná, 2012-2021

A macrorregional Leste apresentou pontos quentes (municípios de maior ocorrência) envolvendo principalmente os municípios da região metropolitana de Curitiba (p-valor<0,001; z-score 4,76) (Figura 3C). A macrorregional Oeste apresentou pontos quentes e frios (municípios de menor ocorrência) com relação aos aglomerados espaciais considerando as taxas de óbitos (Figura 3D). A macrorregional Norte evidenciou pontos quentes, enquanto a Leste mostrou pontos frios. Tais dados apresentaram p-valor de 0,745 e z-score de -0,32.

**Figura 3:** Distribuição espacial dos óbitos e taxa de mortalidade do câncer de mama padronizadas por idade e suas áreas de risco; (A) distribuição espacial dos números de óbitos corrigidos; (B) distribuição espacial das taxas de mortalidade por câncer de mama padronizadas por idade; (C) áreas espaciais de maior ocorrência referentes aos números de óbitos corrigidos por câncer de mama; (D) áreas espaciais de maior ocorrência referentes às taxas de mortalidade por câncer de mama padronizadas por idade. Paraná, 2012-2021

## Discussão

No período 2012-2021, houve maior mortalidade entre mulheres com 50-69 anos, de raça/cor da pele predominantemente branca, sem companheiro e com até 7 anos de estudo, com tendência temporal crescente para todo o Paraná, especialmente para a macrorregional Leste. A distribuição espacial por número de óbitos também identificou pontos quentes (aglomerados de valores altos) na macrorregional Leste. A distribuição por taxas de mortalidade mostrou pontos quentes e frios na macrorregional Oeste, pontos quentes na Norte e pontos frios na Leste.

Foram limitações deste estudo a possível existência de subnotificações no Sistema de Informações sobre Mortalidade. Além disso, há possibilidade de viés quanto à atualização dos dados no sistema ao longo do tempo, após análise dos casos pelas equipes de vigilância epidemiológica. Essas limitações foram minimizadas pelo emprego do fator de correção aplicado neste estudo. Paralelamente, há necessidade de considerar que, no período 2020-2022, afetado pela pandemia da covid-19, ocorreram limitações no rastreamento do câncer de mama, favorecendo que alguns óbitos por covid-19 possam ter sido potencialmente causados pelo câncer de mama em outras circunstâncias.

A taxa de mortalidade por câncer de mama no Paraná foi mais elevada do que em outras regiões do país. Na Bahia, a taxa de mortalidade foi 9,3 óbitos por 100 mil mulheres em 2018 (16). No estado de São Paulo, a taxa estimada para 2021 foi 11,71 óbitos por 100 mil mulheres (4).

A tendência temporal crescente para as taxas de mortalidade por câncer de mama no Paraná também é observada em outros estados brasileiros. Em Santa Catarina, houve crescimento expressivo de 8,38% em 2017 (17). No Ceará, houve aumento anual de 0,43% dos óbitos notificados durante o período 2005-2015 (18). Contudo, o aumento não ocorreu apenas no Brasil. Em 17 países pertencentes à América Latina e ao Caribe no período 1997-2017, houve aumento anual de 2,8% na mortalidade por câncer de mama em mulheres (19).

O aumento da mortalidade por câncer de mama pode estar relacionado com a ineficiência dos serviços de saúde, especificamente na ausência de medidas profiláticas, diagnósticos precoces e realização de tratamentos em fase inicial da doença (18). Entre os maiores problemas enfrentados pela Atenção Primária à Saúde, pode-se citar o subfinanciamento do Sistema Único de Saúde, o congelamento dos gastos e a deterioração dos serviços. Tais questões resultaram em falhas na assistência e, consequentemente, no aumento da mortalidade (20).

Analisando as características demográficas na população estudada, observou-se cenário com tendência crescente e alta mortalidade na faixa etária de 50-69 anos, coincidindo com a população-alvo de rastreamento. Para ocorrer significativas reduções da mortalidade por esse câncer, a Organização Mundial da Saúde estipulou que 70,0% da população feminina pertencente à faixa etária alvo de rastreamento devem realizar a mamografia. No entanto, em 2022, o Brasil rastreou 65,9% dessas mulheres (4), podendo ser fator contributivo para a alta mortalidade e tendência crescente nessa faixa etária.

Notou-se tendência temporal crescente também em mulheres com 70-80 anos e mais de idade. Esse cenário pode ser justificado devido à presença de comorbidades nessa faixa etária, como diabetes, hipertensão arterial, depressão e artrite. Tais comorbidades resultam em menor sobrevida dessas mulheres, pois minimizam a intensidade do tratamento a ser implementado (21).

Apesar de a maior mortalidade ocorrer entre mulheres acima de 50 anos de idade, também ocorrem óbitos entre aquelas com idade de 20-49 anos. Há a necessidade de educação em saúde para a consciência da mulher sobre suas mamas, o exame clínico realizado pelo profissional de saúde e o rastreamento precoce para mulheres mais jovens com histórico familiar da doença (22).

Nesta análise, mulheres de raça/cor da pele branca apresentaram tendência crescente e elevadas taxas de mortalidade, o que pode estar relacionado ao fato de grande parte da população do Paraná (70,32%) identificar-se como branca (23). Embora a raça/cor da pele branca apresente a maior mortalidade, a raça/cor da pele não branca também apresenta crescimento, o que demonstra a vulnerabilidade das mulheres para o câncer de mama independentemente de raça/cor da pele.

Em Sergipe, no período 1996-2017, maiores taxas de mortalidade e tendência temporal crescente foram observadas entre mulheres sem companheiro (24). A situação conjugal não é considerada fator de risco para o câncer de mama, mas entende-se que tal característica é importante para avaliar o perfil social dessas mulheres. Isso ocorre por ser esse um indicador de vulnerabilidade, visto que mulheres sem companheiro têm maior possibilidade de estarem desprovidas do aporte financeiro e emocional do parceiro, o que dificulta o acesso ao diagnóstico e ao tratamento precoce, corroborando a mortalidade por essa causa (25).

As maiores taxas de óbito por câncer de mama e tendência estacionária entre mulheres com até sete anos de estudo também é ratificada por outros achados. No Rio Grande do Sul, entre 1999 e 2019, foi constatado que mulheres com escolaridade inferior a oito anos de estudo apresentaram maiores taxas de mortalidade (26). Dados de 2019 evidenciaram que a baixa escolaridade é uma das características para o diagnóstico em estágios avançados da doença devido à procura tardia por atendimento de saúde (27).

A tendência estacionária dessa variável evidenciou a necessidade de maior atenção a mulheres com escolaridade inferior a sete anos de estudo, utilizando mecanismos que alcancem a compreensão destas e estimulem a realização do rastreamento. Contudo, mulheres com oito e mais anos de estudo apresentaram tendência crescente, o que indicou a necessidade de intensificação do rastreamento oportuno e organizado por meio da mamografia, do diagnóstico precoce e da identificação de sinais e sintomas de alterações mamárias independentemente da escolaridade da mulher (22).

Na análise espacial, a macrorregional Leste apresentou as maiores taxas e tendência temporal crescente, o que pode estar associado ao fato de que essa macrorregional abriga 30,9% dos residentes do estado, sendo a região mais populosa, na qual também são observadas as maiores taxas de mortalidade por doenças crônicas não transmissíveis no Paraná (28). Tais constatações sugerem dificuldades no acesso e na oferta dos serviços de saúde para o enfrentamento dessas doenças, o que pode estar contribuindo para o aumento da mortalidade por câncer de mama nessa região.

Quando se analisa a distribuição espacial (GI*) dos óbitos por câncer de mama, observa-se que a macrorregional Leste apresentou pontos quentes na região metropolitana, na qual também se concentram os municípios com maiores números de óbitos. Essa região abriga Curitiba, capital do Paraná, sede da região metropolitana e área mais populosa do estado (28), justificando a predominância de óbitos. Insere-se neste argumento a grande quantidade de pessoas em regiões periféricas, com dificuldade de acesso aos serviços de saúde e às medidas profiláticas, o que favorece o atraso dos diagnósticos e do tratamento precoce (29).

Quando analisada a distribuição espacial (GI*) das taxas de mortalidade por câncer de mama, pontos quentes foram observados nas macrorregionais Norte e Oeste, indicando concentração de municípios com maiores taxas de mortalidade por câncer de mama. Os municípios que compõem essas macrorregionais possuem índice de desenvolvimento humano municipal de 0,4 a <0,6 e de 0,6 a <0,^8^, considerados médio baixo e médio. Esses parâmetros indicam desigualdade social e econômica, já estabelecidos como dificultadores do acesso da população aos serviços de saúde (28), comprovados neste estudo pelas altas concentrações de municípios localizados nessa macrorregional com as maiores taxas de mortalidade.

Entre 2012 e 2021, houve aumento nos óbitos por câncer de mama no Paraná. Com base na análise de tendência, esse fenômeno foi crescente, em especial para mulheres com idade superior a 50 anos, sem companheiro e independentemente da escolaridade e raça/cor da pele. As principais áreas com tendência de aumento, e consequentemente com maior necessidade de intervenção imediata, envolvem os municípios da macrorregional Leste.

Os serviços de Atenção Primária devem planejar ações de conscientização e oferta de pronto atendimento, inclusive em horários flexíveis que favoreçam o comparecimento da mulher fora do seu horário de trabalho para a realização do exame clínico pelo profissional de saúde. Há necessidade do ensino sobre sinais e sintomas do câncer de mama para promover o autoconhecimento da mulher quanto às possíveis alterações em suas mamas, proporcionando procura imediata para o rastreamento por imagem.

Os resultados deste estudo são importantes para o planejamento e a implementação de ações e políticas públicas de saúde voltadas à prevenção e à detecção precoce do câncer de mama, contribuindo para diminuição da mortalidade por essa causa.

## Data Availability

O banco de dados e os códigos de análise utilizados na pesquisa estão disponíveis em: http://www2.datasus.gov.br/DATASUS/index.php?%20area=0203; e https://www.ibge.gov.br/geociencias/cartas-e-mapas/bases-cartograficas-continuas.html.
